# The Membrane Cholesterol Modulates the Interaction Between 17-βEstradiol and the BK Channel

**DOI:** 10.3389/fphar.2021.687360

**Published:** 2021-06-11

**Authors:** Sara T. Granados, Ramon Latorre, Yolima P. Torres

**Affiliations:** ^1^ Departamento de Nutrición y Bioquímica, Facultad de Ciencias, Pontificia Universidad Javeriana, Bogotá, Colombia; ^2^ Centro Interdisciplinario de Neurociencia de Valparaíso, Facultad de Ciencias, Universidad de Valparaíso, Valparaíso, Chile

**Keywords:** cholesterol (chol), BK channel, slo1, estradiol (17ß-estradiol), membrane cholesterol

## Abstract

BK channels are composed by the pore forming α subunit and, in some tissues, is associated with different accessory β subunits. These proteins modify the biophysical properties of the channel, amplifying the range of BK channel activation according to the physiological context. In the vascular cells, the pore forming BKα subunit is expressed with the β1 subunit, where they play an essential role in the modulation of arterial tone and blood pressure. In eukaryotes, cholesterol is a structural lipid of the cellular membrane. Changes in the ratio of cholesterol content in the plasma membrane (PM) regulates the BK channel activation altering its open probability, and hence, vascular contraction. It has been shown that the estrogen 17β-Estradiol (E2) causes a vasodilator effect in vascular cells, inducing a leftward shift in the V_0.5_ of the GV curve. Here, we evaluate whether changes in the membrane cholesterol concentration modify the effect that E2 induces on the BKα/β1 channel activity. Using binding and electrophysiology assays after cholesterol depletion or enrichment, we show that the cholesterol enrichment significantly decreases the expression of the α subunit, while cholesterol depletion increased the expression of that α subunit. Additionally, we demonstrated that changes in the membrane cholesterol cause the loss of the modulatory effect of E2 on the BKα/β1 channel activity, without affecting the E2 binding to the complex. Our data suggest that changes in membrane cholesterol content could affect channel properties related to the E2 effect on BKα/β1 channel activity. Finally, the results suggest that an optimal membrane cholesterol content is essential for the activation of BK channels through the β1 subunit.

## Introduction

The large-conductance, Ca^2+^- and voltage-activated K^+^ (BK) channel is a transmembrane protein formed by four α subunits (Latorre et al., 2017). The α subunit contains seven transmembrane segments (S0-S6) with the amino terminus exposed to the external medium and the large carboxyl terminus domain, which contains the Ca^2+^ binding sites in the intracellular medium. The α subunit is ubiquitously expressed in mammalian tissues. In some tissues, it can be co-expressed with different β subunits (β1–β4), which modulate the biophysical and pharmacological characteristics of the α subunit (Orio et al., 2002; Torres et al., 2014). In smooth muscle, α subunit is co-expressed with the β1 subunit, which induces an increase in the apparent sensitivity to Ca^2+^ and slows down the channel-gating kinetics (Brenner et al., 2000; Cox and Aldrich, 2000; Orio and Latorre, 2005a). The presence of the BK α/β1 channels prompts membrane hyperpolarization and vasodilatation, which decrease the risk of pathologies associated with vascular tone (Amberg and Santana, 2003; Contreras et al., 2012). Furthermore, BK channels can be modulated by estrogens such as 17β-Estradiol (E2), when α subunit is co-expressed with the β1 subunit (Valverde et al., 1999; De Wet et al., 2006; Granados et al., 2019). E2 increases the open probability of the BK channel (Valverde et al., 1999; De Wet et al., 2006; Granados et al., 2019), which can regulate the vascular tone, causing a protector effect on the vascular tissue (Plüger et al., 2000; Löhn et al., 2001).

In eukaryotes cholesterol distribution in the cellular membrane controls regulatory and signaling processes (Ropero et al., 2003; Luchetti et al., 2020). Changes in the content of cholesterol can affect both the cell membrane structure and the activity of membrane proteins (Levitan et al., 2014). This regulatory effect can be induced by altering the membrane fluidity, trough lipid-protein interactions, or directly through the modulation of the protein (Bolotina et al., 1989; Lecerf and De Lorgeril, 2011; Brignell et al., 2015; Subczynski et al., 2017a). In particular, given that BK channels cluster in microdomains rich in cholesterol, much attention has been given to the effect of this lipid on the BK channel activity (Bolotina et al., 1989; Babiychuk et al., 2004; Lam et al., 2004; Shmygol et al., 2007; Prendergast et al., 2010; Wu and Marx, 2010; Bukiya et al., 2011c; Tajima et al., 2011). In this regard, the results indicate that membrane cholesterol depletion promotes a small increase in BK current and that membrane cholesterol enrichment induces a significant reduction in the BK current density through a decrease in both open probability (Po) and α subunit expression (Bukiya et al., 2008b; Bukiya et al., 2011a; Dopico et al., 2012; Singh et al., 2012; Wu et al., 2013; Dopico and Bukiya, 2014). Although the β1 subunit is necessary for the modulatory effect of cholesterol derivates (Bukiya et al., 2011a; Bukiya et al., 2011b), it has been proposed that cholesterol effects are mediated only through the α subunit (Bukiya et al., 2011a; Singh et al., 2012).

Considering that BK channel activity is modified by changes in membrane cholesterol content (Wu et al., 2013; Singh et al., 2012) and that our previous results suggest that E2 binds to a hydrophobic pocket contained in the second transmembrane domain of the β1 subunit, lying in an interface with the lipid bilayer (Granados et al., 2019), we reasoned that membrane cholesterol content could modify the interaction of the BK channel with molecules such as E2. Here, we expressed α and β1 subunit in HEK 293 cells and after cholesterol enrichment or depletion we measured the binding and the functional effects of E2 in the BK channel activity. We found that E2 binding to the channel is not affected by changes in membrane cholesterol content. However, the modulation of BK channel activity by E2 is lost.

## Methods

### Reagents and Antibodies

Methyl-β-cyclodextrin (MβCD), water-soluble cholesterol (CLR), membrane-impermeant E2-BSA-FITC, and soluble 17β-Estradiol (E2) were purchased from Sigma-Aldrich (St. Louis, MO, United States). Extracellular rabbit anti-MaxiK-α was purchased from Alomone Labs (Jerusalem, Israel) and extracellular rabbit anti-MaxiK-β1 from Novus Biologicals (Littleton, CO, United States). Secondary antibodies anti-rabbit Alexa Fluor 488, 568, and nuclear marker 4ʹ,6-Diamidino-2-Phenylindole, Dihydrochloride (DAPI) were acquired from Invitrogen (Waltham, MA, United States).

### Cell culture and gene transfection

HEK 293 cells were cultured in Dulbecco’s modified Eagle’s medium (DMEM), Gibco (Waltham, MA, United States) supplemented with 10% fetal bovine serum (FBS), Lonza (Basel, Switzerland) at 37°C and 5% CO_2_. Once the cells reached 70% of confluence were transfected using Lipofectamine 2000 (Invitrogen) and human BKα (U11058) or BKβ1 (U25138) harbored in pcDNA3.1, the plasmids were kindly provided by L. Toro (University of California, Los Angeles, CA). Transfection was done with 1.5 μg of the α subunit and 3 μg of the β1 subunit for a combination of both subunits in a 1:2 proportion. Cells used for the expression, binding assays, and ionic current recordings were seeded on 12 mm glass coverslips coated with poly-D-lysine (Sigma-Aldrich).

### Cholesterol enrichment or depletion

Cholesterol depletion or enrichment was done in HEK 293 cells with MβCD or cholesterol-saturated MβCD (MβCD-CLR) with a ratio of 1:10. A cholesterol stock solution was briefly made by dissolving water soluble cholesterol to a 5 mM concentration in serum-free DMEM medium. The tube was sealed and vortexed and incubated for 1 hour in a shaking bath at 37°C. In experiments with cholesterol enrichment or depletion, cells were washed twice with serum-free DMEM medium and then incubated with the medium containing 5 mM MβCD-CLR or 5 mM MβCD for 1 h at 37°C and 5% CO_2_. After the treatment, the cells were washed twice with serum-free DMEM medium and incubated for at least 2 h in serum-free medium while maintaining the enriched or the depleted cholesterol. The cells were then used for the viability, cholesterol quantification, channel expression, E2 binding essays and electrophysiological recordings.

### Cholesterol membrane content quantification

Cholesterol content in HEK 293 cells was determined by fluorometry using the Amplex® Red Cholesterol Assay quantitation kit (ThermoFisher, Oslo, Norway) following the manufacturer’s instruction. Briefly, detached cells were washed twice with PBS 1X at 4°C, incubated for 60 min at−80°C in a lysis solution with 0.5 M K_2_HPO_4_, 0.25 M NaCl, 25 mM cholic acid and 0.5% Triton X-100 at pH 7.4. The cholesterol content was estimated in a colorimetric assay, and the product was measured in a FLUOstar Omega (BMG Labtech) fluorometer with a 530 nm/590 nm filters. The protein content of each sample was determined using a Bicinchoninic Acid protein assay, and the cholesterol content was expressed as μg/mg of protein. Total cholesterol values were normalized with the total protein concentration for each experiment. To determine the magnitude of the change, we compared each condition against the control that was taken as 100%.

### Cellular viability

Once the cells were treated with MβCD or MβCD-CLR, viability assays were made to confirm that the depletion or enrichment of membrane cholesterol did not cause cellular death. Cellular viability was investigated using the tetrazolium salt thiazolyl blue, also termed MTT (methyl-thiazolyl-tetrazolium). MTT produces a yellow aqueous solution that, on reduction by reducing agents present in metabolically active cells, produces a water insoluble violet-blue formazan. The formazan product was extracted with Dimethyl sulfoxide (DMSO) and measured by spectrophotometry, a FLUOstar Omega fluorometer at 592 nm. The amount of MTT formazan is directly proportional to the number of living cells (Stockert et al., 2012). Results were expressed in percentage of viability; control cells were taken as 100% of viability.

### Expression assays

For the immunochemistry assays, we used non-permeabilized labeling for the membrane expression of human BKα (U11058) and human β1 (U25138) subunits. Transfected cells were seeded on 12 mm glass cover slides. We carried out non-permeabilized labeling, according to Bian et al. (Bian et al., 2011). Briefly, live cells were washed twice with PBS at 37°C and incubated for 1 h at 4°C with either MaxiK-α (1:250) or MaxiK-β1 (1:500) antibodies. The cells were washed twice with PBS and incubated with Alexa Fluor 488 conjugated anti-rabbit antibody (1:1,000) for MaxiK-α and Alexa Fluor 568 conjugated anti-rabbit antibody (1:1,000) for MaxiK-β1 for 1 h, followed by washing twice with PBS-FBS, and a fixing step with paraformaldehyde (3%). The nuclei were stained with DAPI (300 nM) for 15 min at room temperature, washed thrice in PBS, mounted with DAKO (Agilent Technologies, Santa Clara, CA, United States) and visualized with a confocal microscope. We quantified the BK channel membrane-expression using non-permeabilized labeling and flow cytometry. Suspended live cells were washed twice with phosphate-buffered saline (PBS) at 37°C, incubated for 1 h at 4°C with either MaxiK-α (Alomone Labs, 1:50) or MaxiK-β1 (Novus, 1:100). Cells were then washed twice with PBS-FBS (2%) and incubated with Alexa Fluor 488 conjugated anti-rabbit antibody (1:300) for 40 min. After another two washes with PBS-FBS, α and β1 expression were verified in a BD Accuri™ C6 flow cytometer (BD Biosciences, San Jose, CA, United States).

### Binding assays

We carried out binding assays as described before (Valverde et al., 1999; Granados et al., 2019). To detect the E2 binding, we used confocal microscopy and quantified the binding with flow cytometry. For the confocal assays, cells seeded on 12 mm glass cover slide were incubated for 15 min with E2-BSA-FITC (10 μM) at 37°C and 5% CO_2_ with DMEM and FBS 10%, washed twice with PBS at 37°and then visualized with a confocal microscope. For the binding quantification assays, we used flow cytometry. Suspended cells were incubated for 15 min with 10 μM of E2-BSA-FITC at 37°C, washed twice with PBS-FBS at 2%, and measured with a BD Accuri™ C6 flow cytometer.

### Image acquisition and analysis

Cells were observed, and images were collected by confocal microscopy using an Eclipse 80i Nikon (Nikon, Tokyo, Japan) with a 408 nm violet, 488 nm-argon, and 543 nm-He-Ne laser diode lines, with a 450/35, 515/30 and 605/75 emission filters. Z-stack parameters were as follows: z-axis slices at ∼1.0 μm intervals with a final Z-stack thickness of ∼15 μm. Images were obtained using a water immersion 60X objective and the EZ-C1 Nikon program. Images were analyzed using ImageJ software (https://imagej.nih.gov/ij/freely available from National Institutes of Health). The conditions, exposures, and the number of sections were identical for a specific experiment.

### Flow cytometry acquisition and analysis

For the flow cytometry quantification assays, each experiment consisted of three technical replicates repeated at least thrice. The run limit of the samples was set to 20,000 gated events with a flow set to 22 μl/min events per sample. Samples were manually re-suspended before each acquisition, and the cells were gated based on the light-scattering properties in the Side and Forward-scatter parameters. HEK293 cells without transfection were used as a control, and fluorescence was detected with the FL1 (530/533 filter) detector using the BD Accuri™ C6 software. All analyses were made using FlowJo® (Tree Star, Inc., Ashland, OR, United States) and normalizing as reported before (Chan et al., 2013). Briefly, we used the Median Fluorescence Intensity (MFI) of each sample and normalized it to the MFI of the HEK293 cells without transfection as a negative control using Eq. 1.

$$nMFI=\frac{MFIsample}{MFIcontrol}$$ (1)

To compare among samples, the Shapiro–Wilk test and the Kruskal–Wallis non-parametric test (*p* < 0.01) were performed using GraphPad Prism 6 (GraphPad Software, Inc., CA, United States).

### Electrophysiology recordings and data analysis

Patch-clamp recordings were carried out in transfected HEK293 cells on 12 mm glass coverslips coated with poly-D-lysine. 24 h after transfection, ion currents were recorded using the inside-out configuration to determine the E2 modulatory effect. Previously, it was suggested that free E2 has the same effect than E2-BSA on BK channel activity (Valverde et al., 1999). This result supports the use of free E2 to analyze the effect of E2 on BK channel activity after modulation of membrane cholesterol content. Symmetrical solutions contained 140 mM KOH, 10 mM HEPES and 2 mM KCl. Ca^+2^ was buffered with 5 mM EGTA (ethylene glycol-bis (β-aminoethyl ether)-N,N,N',N'-tetraacetic acid) to get free Ca^2+^ concentration of ∼20 nM, in order to ensure a maximum effect of E2. (Valverde et al., 1999) showed that BK activation induced by E2 does not change in the range of 56–300 nM Ca^2+^, however, the channel BK α/β1 response to E2 tends to vanish at Ca^2+^ concentration >1 μM (Valverde et al., 1999). All solutions were adjusted to pH 7.4 using methanesulfonic acid. E2 experiments were conducted using the inside-out configuration. Recordings were made in a ∼20 nM Ca^+2^ solution, to which 10 μM E2 solution was then perfused. Currents were elicited by 120 ms pulses at increasing voltages from −80 to 250 mV in 10 mV increments, followed by a step at −60 mV to measure tail currents. Measurements were made before the solution change and from the time of 5 min after, until 30 min. The maximum effect of E2 occurred after 5 min of exposure to E2. During recording, the E2 was applied to the membrane patches with a perfusion system. Borosilicate glass patch pipettes (1B150F-4, World Precision Instruments, Sarasota, FL, United States) with resistances of 2–4 MΩ were pulled in a horizontal pipette puller (Sutter Instruments, Novato, CA, United States) and fire-polished with a microforge (Narishige, Tokyo, Japan). All experiments were performed at room temperature (22–24°C) using an EPC7 patch-clamp amplifier (HEKA, Lambrecht/Pfalz, Germany). The acquisition software was developed by Dr Patricio Orio using the LabView programming environment (National Instruments, Austin, TX, United States) (Orio and Latorre, 2005b).

All data analyses were performed with Clampfit 10 (Axon Instruments) and Excel 2013 (Microsoft, Redmont, WA, United States). G-V relationships were fitted using a Boltzmann function: G/Gmax = 1/(1 − exp (−zF(V −V_0,5_)/RT), where Gmax is the maximum tail conductance, z is the voltage dependency of activation and V_0.5_ is the half-activation voltage. Gmax, V_0.5_, and z were determined by using the solver complement of Microsoft Excel. Data were aligned by shifting them along the voltage axis by the mean ΔV_0.5_ = (<V_0.5_> − V_0.5_), then binning them in a range of 10 mV, between −80 mV and up to 250 mV. Data was analyzed with a non-parametric *t*-test (*p* < 0.05) using GraphPad Prism 6.

## Results

### Modulation of Cellular Membrane Cholesterol Content Affects the Surface Expression of the α Subunit but Not the β1 Subunit Expression

**FIGURE 1:**
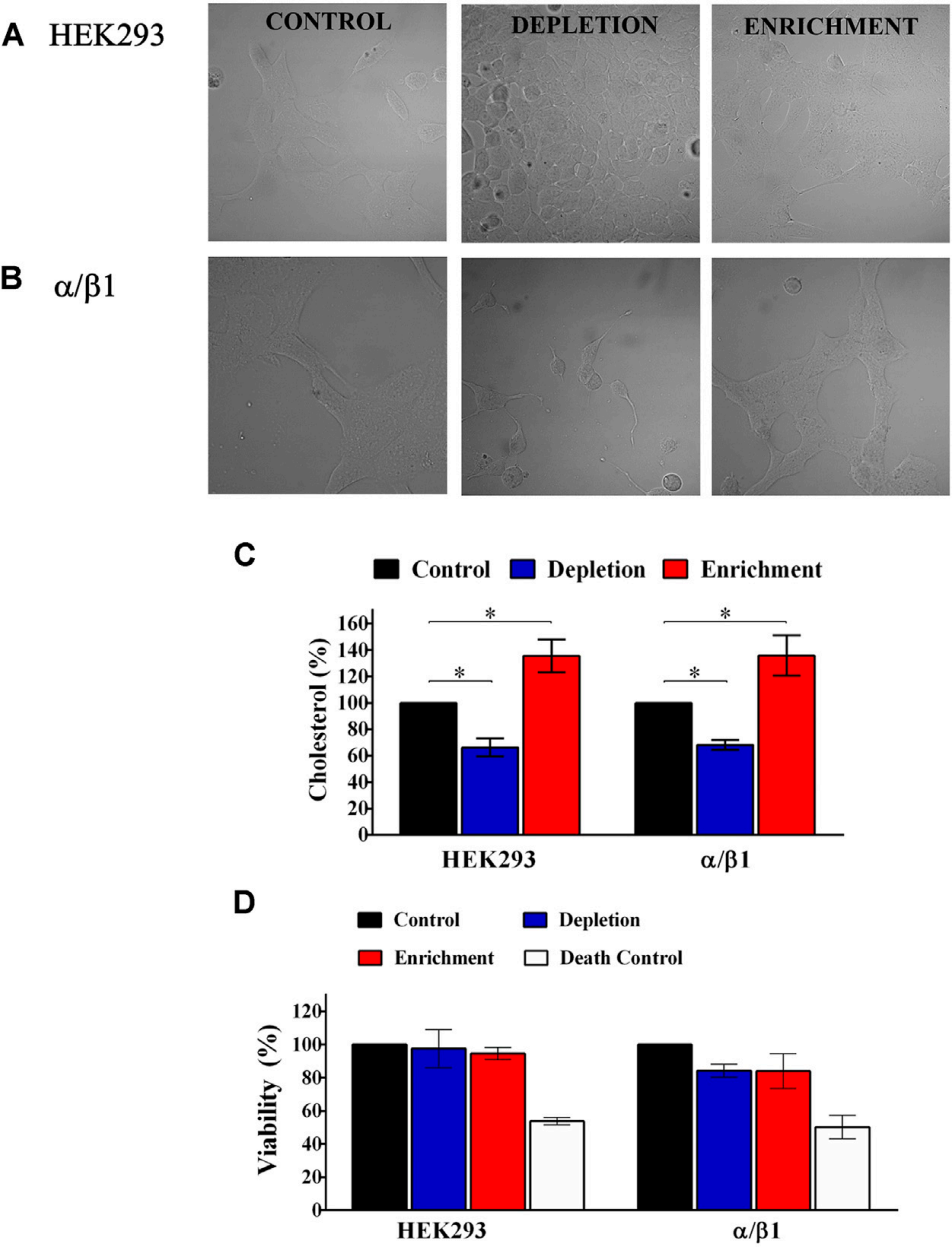
Cholesterol quantification and cell viability of HEK293 control and cells transfected with BK subunits. **(A)** HEK293 cells without transfection. **(B)** HEK293 cells transfected with α+β1. Left panel, control without treatment. Middle panel, Cells treated with 5 mM of MβCD. Right panel, cells treated with 5 mM of CHO-MβCD complex. **(C)** Results for each treatment with MβCD or MβCD-CLR were normalized using the amount of cholesterol contained in cells without treatment (% of control). Control cell (black bars). HEK293 without transfection and HEK293 transfected with α/β1: 100%; Depleted cells (blue bars). HEK293 without transfection: 66.3 ± 6.7; α/β1: 68.2 ± 3.8. Enriched cells (red bars). HEK293: 135.5 ± 12.5; α/β1: 135.8 ± 15.3. Error bars: SEM. *p < 0.05. *n* = 4. **(D)** Percentage (%) of viable transfected cells when membrane cholesterol content has been modified. Control cell (black bars). HEK293 without transfection and HEK293 transfected with α/β1: 100%; Depleted cells (blue bars). HEK293 without transfection: 97.6 ± 11.6; α/β1: 84.2 ± 4. Enriched cells (red bars). HEK293: 94.7 ± 3.7; α/β1: 84 ± 10.5. White bars Death Control represent death cells control with rotenone 50–56%. Error bars: SEM. *n* = 3.

To analyze the effect of CLR in the channel properties, we induced cholesterol depletion or enrichment by treating HEK 293 cells with MβCD or MβCD-CRL complexes, respectively. We observed that treatment with MβCD decreased cell membrane cholesterol between 30 and 40% (from 100 to 70–60%; *n* = 4; *p* ≤ 0.05). Conversely, the treatment with the MβCD-CRL complexes caused a cellular cholesterol enrichment of approximately 40% (from 100 to 140%; *n* = 3; *p* ≤ 0.05), (Figures 1A–C). These effects were similar in all evaluated cells: non-transfected HEK 293 cells and BKα/β1-HEK 293 cells.

We assessed, if changes in membrane cholesterol content affected cell viability and we found that it was not affected after cholesterol depletion (HEK 293: from 100 to 97.6% ± 11.6; BKα/β1-HEK 293: 100 to 84.2% ± 4) or enrichment (HEK 293: from 100 to 94.7% ± 3.7; BKα/β1-HEK 293: 100 to 84% ± 10.5) in any of the evaluated cells (Figure 1D). Rotenone, a mitochondrial respiratory chain inhibitor used as a cell death control, decreased the cell viability in approximately 50% in all cases.

**FIGURE 2:**
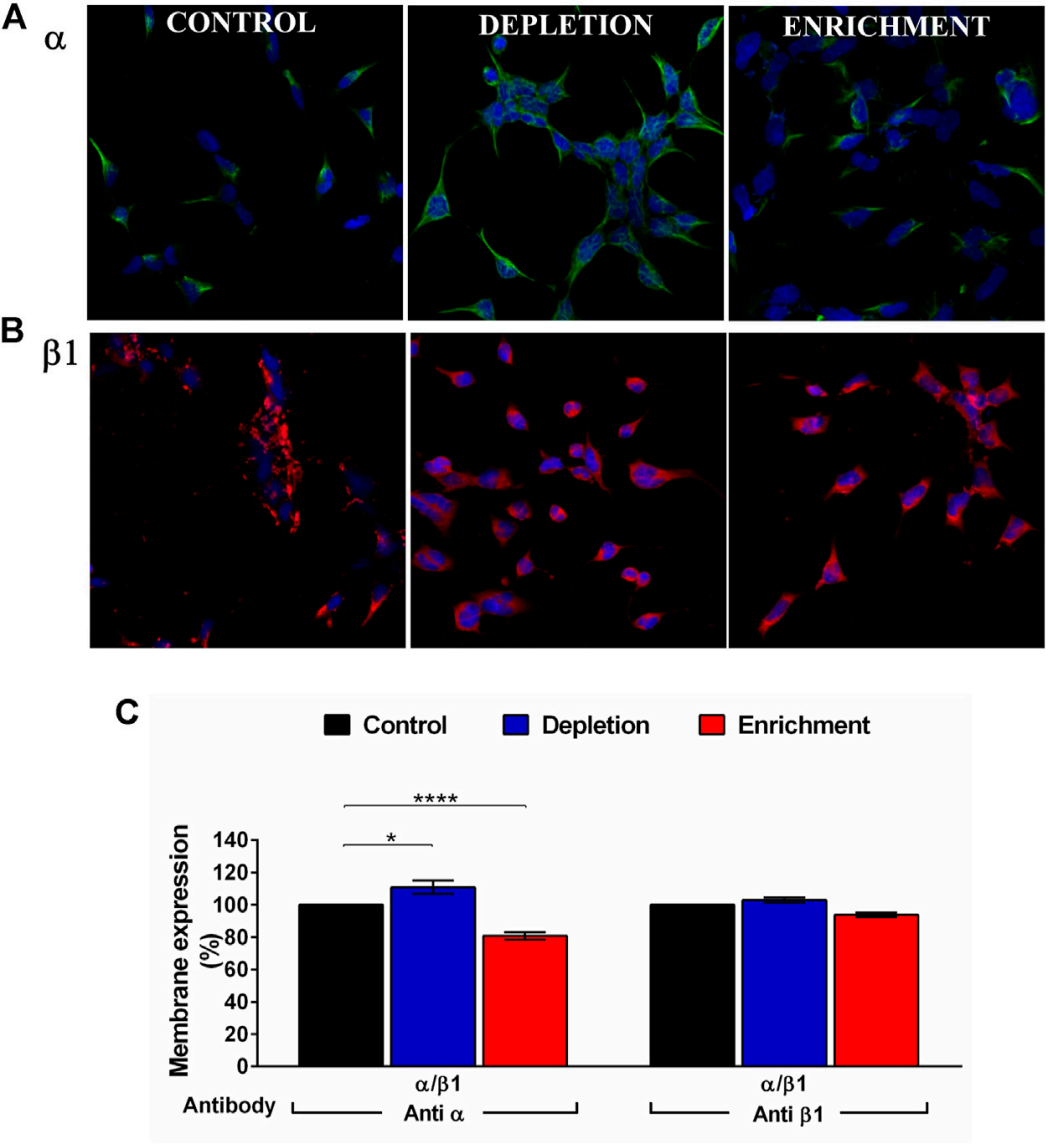
BK channel expression after membrane cholesterol modulation on the HEK 293 transfected cells. **(A)** Immunofluorescence in cells expressing BKα subunit. **(B)** Immunofluorescence on cells expressing β1 subunit. Green: Anti BKα and Alexa Fluor 488 antibodies. Red: Anti BKβ1 and Alexa Fluor 594. Blue: DAPI nuclear stain. **(C)** Expression (% of cells) of BK channel subunits in HEK293 cells transfected with α and β1 subunits using flow cytometry. The blue bars represent cholesterol depleted cells; α:110.9% ± 4.1, β1:102.9% ± 1.7. The red bars are enriched cells. α:80.8% ± 2.3, β1:93.8% ± 1.4. Black bars represent control cells without treatment. 100%. Error bars: SEM. *p < 0.05, ****p < 0.0001. *n* = 5.

When we expressed the α/β1 complexes we found that cholesterol enrichment (Figure 2) significantly decreases the expression of the α subunit (from 100 to 80.8% ± 2.3) while cholesterol depletion increased the expression of that subunit (from 100 to 110.9% ± 4.1). We could not observe any significant change in the expression of the β1 subunit after cholesterol depletion (from 100 to 102.9% ± 1.7) or enrichment (from 100 to 93.8 ± 1.4%) (Figure 2). In agreement with a previous communication (Wu et al., 2013), these results suggest that the α subunit expression is modulated by membrane cholesterol content. α:β subunits stoichiometry is 1:1, hence four α subunits co-assemble with four β subunits (Knaus et al., 1994). However, channels with less β subunits could be expressed, affecting channel properties. It has been reported that channels with less than four subunits show gating properties that scale with the average number of β subunits per channel (Wang et al., 2002). Considering that cholesterol depletion increased the α subunit expression without changes in the expression of β1 subunit, we wonder if α:β1 stoichiometry was modified. To verify that, we performed a voltage protocol to investigate the activation kinetics on the α/β1 channels without any treatment (control) and we compared it with the ones treated with MβCD (depleted) or MβCD-CLR (enriched). We found that the activation kinetics of the treated channels did not change, and it was the same as the control (Supplementary Figure S1). This result shows first, that the 1:1 stoichiometry was not affected when membrane cholesterol was changed and second, that α/β1 complexes was functionally assembled (Bao and Cox, 2005).

### Membrane Cholesterol Content Does Not Affect the E2 Binding to the BK Channel

**FIGURE 3:**
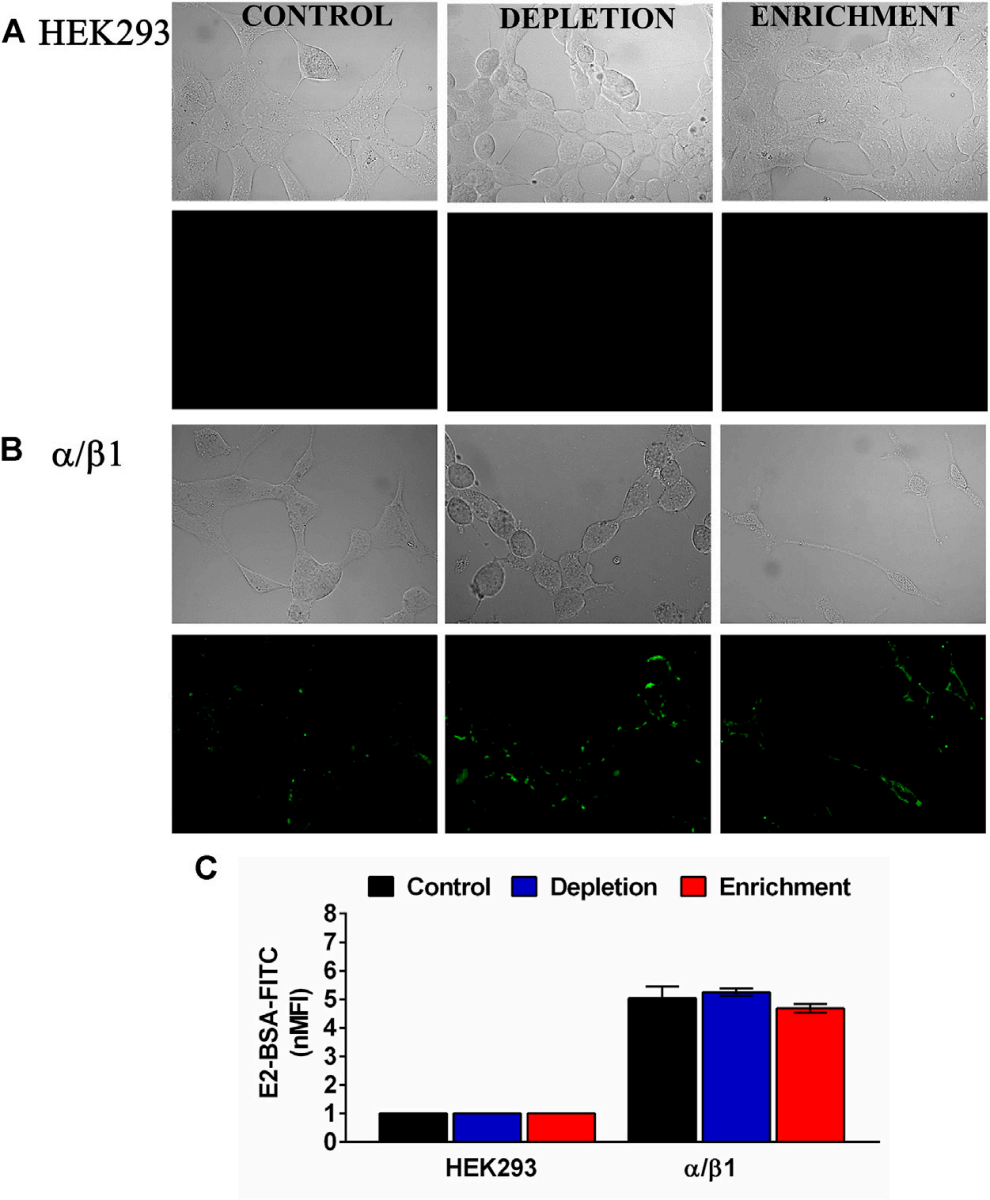
Membrane cholesterol content does not modify E2 binding to the BK channels subunits in HEK293 cells. **(A)** HEK293 cells without transfection. **(B)** α/β1 subunit co- transfection. **(C)** Quantification by flow cytometry of E2-BSA-FITC binding to the BK channel transfected cells. Data were normalized against HEK293 cells without transfection. Depleted cells (blue bars). α/β1:5.2 ± 0.13. Enriched cells (red bars). α/β1:4.7 ± 0.2; black bars represent control untreated cells. α/β1:5.0 ± 0.4. Error bars: SEM. *n* = 3.

To establish the effect of membrane cholesterol content in E2 binding, we studied the binding characteristics of E2 to the BKα/β1 when the membrane cholesterol was depleted or augmented. We used E2-binding assays with a membrane-impermeant conjugate of fluorescein isothiocyanate-labeled E2 covalently linked to albumin (E2-BSA-FITC). Nonetheless the observed changes in the expression of BK α subunit, neither in cholesterol depletion nor enrichment we observe changes in E2 binding to BKα/β1 (Figures 3A–C). These results suggest that membrane cholesterol content does not affect the E2 binding to BK channel. The observed is in accordance with our findings about no changes in the β1 subunit expression after changes in membrane cholesterol concentration.

### Changes in Membrane Cholesterol Content Induce the Functional Loss of E2 on the α/β1 Complexes

**FIGURE 4:**
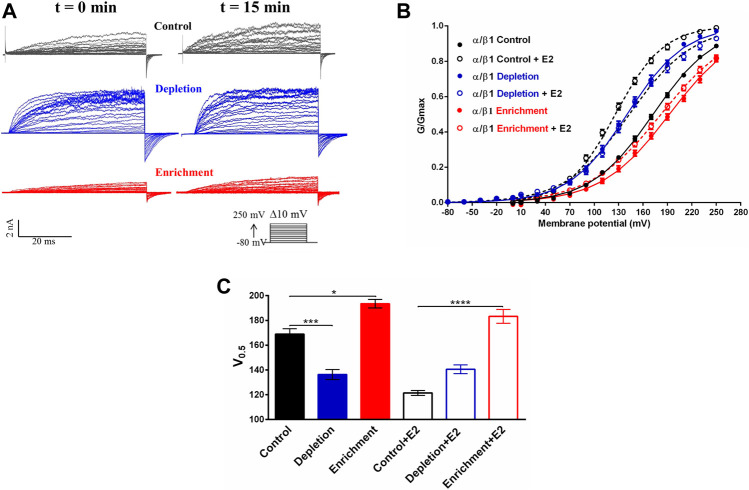
Effect of the membrane cholesterol content on the modulation of the BK channel by E2. **(A)** Representative recordings of BKα/β1 channel current before and after 10 μM E2. Black traces: control without treatment; blue traces: depletion with MβCD; red traces: enrichment with MβCD-CLR. **(B)** G/V relationships for α/β1 channels complexes before (filled circles) and after exposition to 10 μM E2 (empty circles). Lines represent the best Boltzmann fit. Control: black symbols; depletion: blue symbols; enrichment: red symbols. **(C)** Quantification of V_0.5_ obtained from the G-V curves from α/β1 channels. Black traces: control without treatment; blue traces: depletion with MβCD; red traces: enrichment with MβCD-CLR, with or without E2 as indicated (mean ± SEM). Differences between channels and experimental conditions were analyzed using a *t*-test. Half activation voltages (V_0.5_) were: Control conditions**:** 169 ± 4 mV (black filled bar), Control conditions + E2**:**121 ± 2 mV (black empty bar); cholesterol depletion: 137 ± 4 mV (blue filled bar), cholesterol depletion + E2: 140 ± 4 mV (blue empty bar); cholesterol enrichment: 194 ± 3 mV (red filled bar), cholesterol enrichment + E2: 187 ± 5 mV (red empty bar). ****p < 0.0001, ***p < 0.001, *p < 0.05. *n* = 6.

To evaluate if changes in the membrane cholesterol content affect the modulation that E2 induces in BK channel activity, we evaluate the effect of E2 in biophysical properties of BKα/β1 channel expressed in cells after cholesterol depletion or enrichment. Electrophysiology assays was performed at 20 nM calcium to ensure a maximum effect of E2 since it was previously reported that the BK channel response to E2 tends to vanish at Ca^2+^ concentration >1 μM. Previous reports suggest that free E2 has the same effect than E2-BSA on BK channel activity, both in heterologous and endogenous expressed channels (Valverde et al., 1999). Considering these results, it is reasonable to assume that the E2 bound to BSA has similar properties to free E2 and the effect induced by changes in membrane cholesterol content could be comparable in both E2-BSA and free E2. Hence, we used free E2 to analyze the effect of E2 on BK channel activity after modulation of membrane cholesterol content. Confirming previous results (Wu et al., 2013), we found that membrane cholesterol depletion caused a leftward shift in the conductance-voltage curve of the BKα/β1 channel (V_0.5_ changes from 169 ± 4 to 137 ± 4 mV; *n* = 6; *p* = 0.001) (Figure 4). Conversely, membrane cholesterol enrichment caused a rightward shift of the conductance-voltage curve (V_0.5_ changed from 169 ± 4 to 194 ± 3 mV (Figure 4).

In control conditions, 10 μM E2 induced a leftward shift in the V_0.5_ of the GV curve of 50 ± mV in cells expressing BKα/β1 complexes (Figure 4) (Valverde et al., 1999; Granados et al., 2019). Interestingly, we did not observe any effect of E2 in the BK channel activity, after cholesterol depletion. Furthermore, our results showed that cholesterol depletion prevents the E2 from having a modulatory effect on the channel since the macroscopic currents and the V_0.5_ remains the same 15 min after the addition of E2 (from 137 ± 4 mV to 140 ± 4 mV after E2 treatment) (Figure 4C). That is an interesting result considering that the binding assays (Figure 3) showed that E2 binds to the BKα/β1 complexes after cholesterol depletion. Similar behavior was observed after cholesterol enrichment, where we did not observe a significant change in BK channel V_0.5_ after E2 treatment (194 ± 3mV to 187 ± 5 mV) (Figure 4C).

These results showed that changes in the membrane cholesterol cause the loss of the modulatory effect of E2 on the BKα/β1 channel activity. Considering that E2 binding is not affected after cholesterol depletion or enrichment, the results suggest that changes in membrane cholesterol content could affect channel properties related to the E2 effect on BK channel activity.

Membrane cholesterol depletion causes an increment in both BKα channel expression and the evoked current of the channel, a decreased V_0.5_ causing a left shift of the G-V curves, and, most importantly, a loss on the E2 modulatory effect on the BK channel, but not the binding. Although cholesterol enrichment causes the opposite effect on the channel expression and a rightward shift of the G-V curves, the modulatory effect of E2 is lost without changes in binding.

## Discussion

In this study, we investigated the effect of the membrane cholesterol on the interaction of E2 with the BK channel. We found that modifications in membrane cholesterol content eliminate the BK channel activity modulation induced by E2, without changes in the E2 binding to the channel. 17β-Estradiol is a steroid hormone involved in diverse physiological functions, including a protective effect against cardiovascular diseases (CVD) (Gao and Dahlman-Wright, 2011). In order to induce their effects, E2 must cross the plasma membrane to interact with intracellular receptors or bind to membrane steroid receptors and ion channels (Gao and Dahlman-Wright, 2011; Atkovska et al., 2018). In that way, it is relevant to the understanding of estradiol-membrane interactions and how changes in plasma membrane properties can affect this interaction. It has been reported that cholesterol affects the interaction of diverse molecules with plasma membrane by different mechanisms, including changes in physical properties of the membrane and modifying specific interactions with membrane proteins (Wennberg et al., 2012; Subczynski et al., 2017a). There are many reports about the effect of cholesterol in ion channels activity. In TRPA1 channels, cholesterol depletion decreases the channel activation after the addition of allyl isothiocyanate (AITC) (Startek et al., 2019). It has also been reported that calcium-activated chloride channels activity is affected by cholesterol depletion (Sones et al., 2010). In both cases, mechanisms involved the presence of the channels in lipid rafts.

BK channels expression and activity are also modulated by cholesterol, and the reports suggest tissue and cell-specific effects (Wu et al., 2013). Cholesterol play an important role in the formation of membrane microdomains and it is known that BK channels cluster in them (Tajima et al., 2011). So, changes in membrane cholesterol content could be important to regulate the channel expression. Tajima et al. suggested that cholesterol enrichment does not change the overall surface density of the channels, but induce a change in channel distribution, increasing the proportion of BK channels in lipid rafts (Tajima et al., 2011). Additional reports showed that cholesterol depletion promotes a redistribution of the BK channel to non-caveolar fractions by raft disruption (Wang et al., 2005; Riddle et al., 2011). Here, we observed that changes in membrane cholesterol content modify the expression of the α subunit (Figure 1). However, the expression of the β1 subunit was not modified. Wu and cols (2013) reported similar results and suggested that the β1 subunit has an important role in the regulation of the BK α subunit expression by cholesterol, in a mechanism that involve the channel protein degradation mediated by proteasome and lysosome pathways. It is interesting to note that the β1 subunit is not affected by the degradation system in cells with cholesterol enrichment (Wu et al., 2013).

Previously, we reported that E2 binds to a hydrophobic pocket at the β1 subunit through π -stacking and Van der Waals interactions in the TM2. The binding site of E2 includes W163 and F166 residues in β1 subunit which are in contact with the lipid bilayer (Granados et al., 2019). Here, we were interested in determining if the E2-BK channel interaction was affected by changes in the lipid bilayer composition derived of modifications in the membrane cholesterol content. Our results did not show any variation in the E2-BSA-FITC binding to the BK channel after changes in membrane cholesterol content. This is an expected result, considering that binding site for E2 is in the BK channel β1 subunit, and the expression of this subunit was not affected by cholesterol modulation. Moreover, we demonstrated that expressed channels was composed but both α and β subunits. Even though binding of E2-BSA to BKα/β1 does not depend on membrane cholesterol content, we did not find any change in channel activity after free E2 addition in cells where membrane cholesterol content was enriched or depleted.

There is a large set of evidence that increases in membrane cholesterol regulate membrane fluidity and rigidity, affecting the channel activity and the response to diverse molecules (Leichleiter et al., 1986; Wennberg et al., 2012; Subczynski et al., 2017b). Furthermore, as other ion channels, BK channels have cholesterol recognition regions (CRAC) that are involved in changes in channel activity (Dopico and Bukiya, 2014). Cholesterol enrichment also increases the proportion of BK channels in lipid rafts, where their activity is decreased. All these mechanisms could be involved in the changes observed in E2-BK channel interaction after cholesterol enrichment. Cholesterol depletion increases the BK channel activity by a mechanism related to the channel's expression in lipid rafts and decreasing membrane cholesterol reduces the presence of BK channels in lipid rafts and induce a higher activity of the channel (Wang et al., 2005; Riddle et al., 2011). Contrary to expectations, the effect of E2 on the BK channel was abolished when the membrane cholesterol was decreased or after cholesterol enrichment. Our results suggest that an optimal membrane cholesterol concentration is essential for the appropriate E2-BK channel interaction. Similar effects have been previously reported in Kv channels, where both cholesterol depletion or enrichment promoted the channel inhibition. Delgado-Ramirez and cols (2018) suggested that membrane cholesterol changes induce both, a disturbance of the interaction between cholesterol and the Kv channels and changes in the plasma membrane physical properties (Delgado-Ramírez et al., 2018).

Previous to the interaction with their receptor, steroids must partitioning into the plasma membrane (Atkovska et al., 2018), so some reports suggest an important role of the partitioning coefficient in the physiological function of molecules like estradiol (Heerklotz and Keller, 2013). Changes in the membrane cholesterol content induce modifications in partition coefficient of estrogens (Yamamoto and Liljestrand, 2004) which could decrease the steroid content in the plasma membrane and reduce the steroid binding to the BK channel. Similar effects were reported with ethanol (EtOH), where it was observed an essential role of their partitioning into lipid membranes to reach the sites of action (Bukiya et al., 2014). Cholesterol enrichment decreases the ethanol partition into the membrane and induces a change in the effect that ethanol has on the BK channel activity (Bukiya et al., 2012; Bisen et al., 2016). Alterations in the interaction of TRPV1 with capsaicin analogs were also associated with changes in the lipid partition coefficient, which induced an increase in the half-maximal effective concentration (EC_50_) (Yang et al., 2015). Nonetheless, considering that we did not observe any change in E2-BSA-FITC binding to the channel, we suggest that eventual changes in partition coefficient induced by modifications on membrane cholesterol, are not responsible for the observed loss of E2 effect on the BK channel.

The final effect of an agonist depends of both, the binding affinity to the receptor and the energy efficiency, which promote the conformational changes for receptor activation and gating (Nayak et al., 2019) (Tripathy et al., 2019). In membrane receptors such as Acetylcholine receptor (AChR) it has been reported that the binding of an agonist decreases the energy barrier between close and open and stabilizes the open conformation (Nayak et al., 2019). Considering our results about the loss of E2 effect on the BK channel activity without affect the binding, it is feasible that changes in membrane cholesterol content could modify the energy efficiency and affect the stabilization of BK conformational changes, related with the increase on the channel activity. It is known that steroids must get an specific orientation and insertion depth in the membrane to induce the activation of the membrane receptors (Atkovska et al., 2018). For example, pregnenolone sulfate and pregnenolone acetate are steroids with different effect on TRPM3 activity. The first molecule is an agonist meanwhile the other does not have any effect on the channel. The different effect has been related with the orientation of the molecules in the plasma membrane where unfavorably oriented molecules could loss the modulation of the channel activity (Atkovska et al., 2018). Lithocholate (LC) is other steroid derivate which activate BK channel through binding to β1 subunit, similar to reported for E2 (Bukiya et al., 2008b). Changes in structural characteristics of LC, such as C3 hydroxy orientation or cis-trans configuration, affect the BK channel activation (Bukiya et al., 2008a) suggesting a specific membrane orientation to induce the reported effects on BK channel activity. We suggest that changes in membrane cholesterol content induce changes in the E2 binding site orientation, which affect the energy required for induce changes in BK channel gating derived from free E2 modulation without affect the E2-BSA-FITC binding. However, it is necessary to do more experiments to determine the specific mechanism involved in the observed effect.

In conclusion, we report that cholesterol depletion and enrichment induce the loss of the modulatory effect of E2 on the BK channel, without changes in the E2 binding. We previously reported that the effect of E2 on BKα/β1 involves an increase in the equilibrium constant that defines the coupling of voltage sensor with the pore. From our results, we speculate that although changes in membrane cholesterol content do not affect the E2-BSA-FITC binding, they could affect the allosteric coupling between the voltage sensor and the pore induced by E2. Nevertheless, we need to carry on more experiments to explore the proposed suggestion. Furthermore, in addition to its importance in regulating the membrane properties, cholesterol is an essential lipid in the cellular metabolism and precursor of steroid hormones (Arnold and Kwiterovich, 2004; Goluszko and Nowicki, 2005; Lecerf and De Lorgeril, 2011). Because of its metabolic importance, cholesterol homeostasis is a highly regulated process in cells, and a disruption in that regulation is usually the cause of several pathologies. Alterations in cholesterol content in humans are related to the development of cardiovascular diseases (Dogan et al., 2019) (Bukiya and Dopico, 2019). Considering the physiological importance of cholesterol, our data contribute to understanding the effects of cholesterol homeostasis in the 17β-estradiol effect.

## Data Availability

The raw data supporting the conclusion of this article will be made available by the authors, without undue reservation.
