# Supplementary material for: The Membrane Cholesterol Modulates the Interaction Between 17-βEstradiol and the BK Channel
Source: Front Pharmacol. 2021 Jun 11;12:687360. doi: 10.3389/fphar.2021.687360 (PMC8226216; doi:10.3389/fphar.2021.687360)
Supplement: Supplementary file 1 [file Image1.pdf]

Supplementary Material

Supplementary Figures

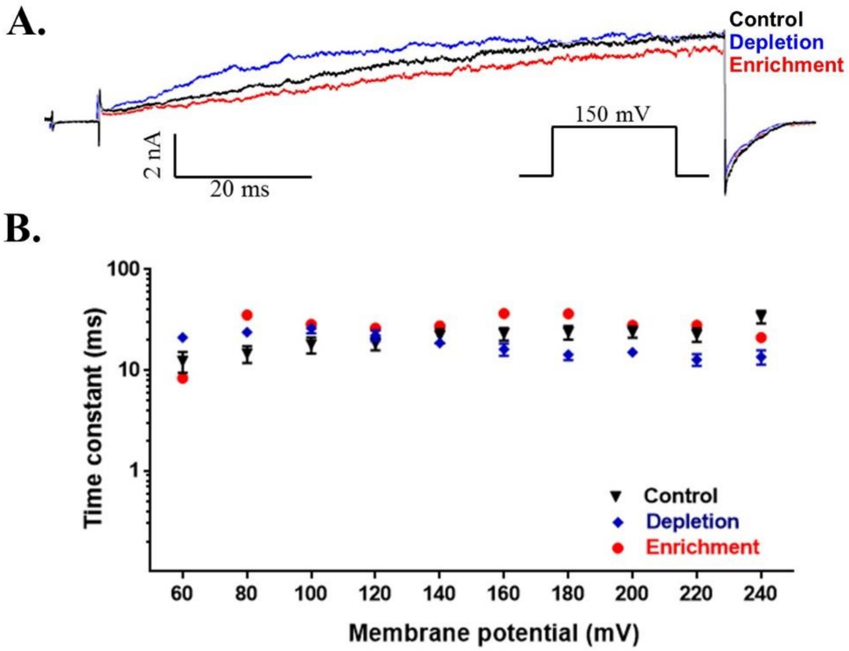

Supplementary Figure 1. Effect of the cholesterol membrane modulation on the BKα/β1 channel macroscopic kinetics. **(A)** Representative macroscopic current records at 150 mV of α/β1 channels expressed in HEK293 cells without cholesterol treatment (Control) (black traces), depleted with MβCD (blue traces) and enrichment with MβCD-CLR (red traces). **(B)** Activation time constant (ms) plotted against applied voltage. α/β1 channels expressed in HEK293 cells without treatment (triangle) and treated with MβCD (rhombus), or MβCD -CLR (circle). n=5-7. Symbols represent mean ± SEM.
